# DCC expression is related to mucinous differentiation but not changes in expression of p21^WAF1/Cip1^ and p27^Kip1^, apoptosis, cell proliferation and human papillomavirus infection in uterine cervical adenocarcinomas

**DOI:** 10.1038/sj.bjc.6690320

**Published:** 1999-04

**Authors:** M Saegusa, I Okayasu

**Affiliations:** Department of Pathology, Kitasato University School of Medicine, 1-15-1 Kitasato, Sagamihara, Kanagawa 228-8555, Japan

**Keywords:** uterine cervix, adenocarcinoma, DCC, p21^WAF1/Cip1^, p27^Kip1^, apoptosis

## Abstract

To clarify possible roles of DCC expression in tumour differentiation and cell kinetics, we immunohistochemically investigated 80 uterine cervical adenocarcinomas (C-ACs), including 31 mucinous (M) and 31 endometrioid (E) lesions, and 18 adenocarcinomas in situ (AIS), along with 39 normal cervical samples. The results were compared with findings for p21^WAF1/Cip1^ and p27^Kip1^ expression, apoptosis, cell proliferation and human papillomavirus (HPV) infection. Nine C-AC cases were also examined using a combination of the reverse transcription-polymerase chain reaction and Southern blot hybridization, as well as Western blot assays. Significantly decreased DCC scores were observed in E-ACs but not M-ACs, as compared to normal cervical glandular epithelia and AIS. Average p21^WAF1/Cip1^ and p27^Kip1^ scores were significantly higher in E-ACs than M-ACs, in line with high apoptotic, mitotic and Ki-67 labelling indices. A concordance of the results for DCC and p21^WAF1/Cip1^ expression between mRNA- and protein-based assays was also noted. Change of DCC expression, however, was not related to any of the cell kinetic markers or clinicopathological features in ACs of either type. There was also no association with the HPV status, although infection was significantly linked with high values for cell kinetics. These results suggest that DCC expression in C-ACs is closely associated with mucinous differentiation. © 1999 Cancer Research Campaign


*British Journal of Cancer* (1999) **80**(1/2), 51–58
© 1999 Cancer Research Campaign
Article no. bjoc.1998.0320

### DCC expression is related to mucinous differentiation but not changes in expression of p21$^{WAF1/Cip1}$ and p27$^{Kip1}$, apoptosis, cell proliferation and human papillomavirus infection in uterine cervical adenocarcinomas

**M Saegusa and I Okayasu**

Department of Pathology, Kitasato University School of Medicine, 1-15-1 Kitasato, Sagamihara, Kanagawa 228-8555, Japan

**Summary** To clarify possible roles of DCC expression in tumour differentiation and cell kinetics, we immunohistochemically investigated 80 uterine cervical adenocarcinomas (C-ACs), including 31 mucinous (M) and 31 endometrioid (E) lesions, and 18 adenocarcinomas in situ (AIS), along with 39 normal cervical samples. The results were compared with findings for p21$^{WAF1/Cip1}$ and p27$^{Kip1}$ expression, apoptosis, cell proliferation and human papillomavirus (HPV) infection. Nine C-AC cases were also examined using a combination of the reverse transcription-polymerase chain reaction and Southern blot hybridization, as well as Western blot assays. Significantly decreased DCC scores were observed in E-ACs but not M-ACs, as compared to normal cervical glandular epithelia and AIS. Average p21$^{WAF1/Cip1}$ and p27$^{Kip1}$ scores were significantly higher in E-ACs than M-ACs, in line with high apoptotic, mitotic and Ki-67 labelling indices. A concordance of the results for DCC and p21$^{WAF1/Cip1}$ expression between mRNA- and protein-based assays was also noted. Change of DCC expression, however, was not related to any of the cell kinetic markers or clinicopathological features in ACs of either type. There was also no association with the HPV status, although infection was significantly linked with high values for cell kinetics. These results suggest that DCC expression in C-ACs is closely associated with mucinous differentiation.

**Keywords:** uterine cervix; adenocarcinoma; DCC; p21$^{WAF1/Cip1}$; p27$^{Kip1}$; apoptosis

Uterine cervical adenocarcinomas (C-ACs) are important neoplasias in the female reproductive tract, along with squamous cell carcinomas (SCCs). Several studies have demonstrated possible associations between gene abnormalities and tumorigenesis of SCCs (Park et al, 1994; Saegusa et al, 1995), but there have been only a few reports regarding the molecular mechanisms of development and progression of C-ACs.

Early studies of tumour suppressor genes indicated that their gene products may be involved in preventing cellular proliferation, possibly directly through inhibiting DNA replication or indirectly through regulating the transcription of other genes involved in controlling the cell cycle (Ginsberg et al, 1991; Mercer et al, 1991). The DCC gene, located in the chromosome 18q21.3 band, was originally identified as a tumour suppressor gene in colorectal carcinomas (Fearon et al, 1990). Klingelhutz et al (1995) have demonstrated that a full length DCC can inhibit tumorigenicity of neoplastic cells lacking DCC expression. Recent studies have revealed that DCC gene alterations are also involved in tumorigenesis in a variety of other human malignancies (Huang et al, 1992; Uchino et al, 1992; Gao et al, 1993). Although frequent loss or reduction of DCC expression has been reported in endometrial and ovarian carcinomas (Gima et al, 1994; Enomoto et al, 1995), alteration of this gene in their AC counterparts in the cervix has not been described in detail.

*Received 16 June 1998*
*Revised 21 August 1998*
*Accepted 26 August 1998*

*Correspondence to:* M Saegusa

Analysis of tissue kinetics, including cell deletion (apoptosis) and proliferation, provides information on the mechanisms of tumour progression, since loss of tissue homeostasis is closely linked to tumour growth patterns. Recently, multiple cyclins and cyclin-dependent kinases (CDKs) as positive cell cycle regulators and specific inhibitory proteins (CDIs), including p21$^{WAF1/Cip1}$, p27$^{Kip1}$, p16$^{MTS1}$ and p15$^{MTS2}$, have been identified in mammalian cells. CDIs have been proposed as candidate tumour suppressor genes, since their overexpression is associated with G1 arrest (El-Deiry et al, 1993; Serrano et al, 1993; Harper et al, 1993; Hannon and Beach, 1994; Kamb et al, 1994; Nobori et al, 1994; Polyak et al, 1994; Toyoshima and Hunter, 1994).

In the present study, we immunohistochemically investigated normal and malignant cervical glandular components to clarify the relation of DCC expression to tumour differentiation and cell kinetics. In addition, a combination of the reverse transcription-polymerase chain reaction (RT-PCR) and Southern blot hybridization (SBH), and Western blot assays were performed. Expression of p21$^{WAF1/Cip1}$ and p27$^{Kip1}$, apoptosis, cell proliferation and human papillomavirus (HPV) infection were selected as parameters for comparison.

### MATERIALS AND METHODS

### Cases

A total of 80 cases of C-ACs, surgically resected at the Kitasato University Hospital from 1985 to 1997, were investigated, along with 39 samples of normal uterine cervix adjacent to carcinomas. All tissues were routinely fixed in 10% formalin and embedded in paraffin wax. Histological diagnosis was performed according to the World Health Organization (WHO) classification. Carcinoma cases comprised 31 endometrioid (E-AC, 17 grade (G) 1, and 14 G2 and G3), 31 mucinous (M-AC, 19 G1, and 12 G2 and G3), and 18 adenocarcinoma in situ (AIS) lesions. Of the 31 E-ACs, 20 were classified as FIGO (International Federation of Gynecology and Obstetrics) stage I, 11 as stage II–IV, and eight were positive for lymph node metastasis. Of the M-ACs, 20 were stage I and 11 stage II–IV, while nine were positive for lymph node metastasis. Nine C-ACs (two M- and seven E-ACs) were snap-frozen in liquid nitrogen for RT-PCR/SBH analysis of DCC and $p21^{WAF1/Cip1}$ mRNAs, and Western blot assays for $p21^{WAF1/Cip1}$ and $p27^{Kip1}$ proteins.

### Immunohistochemistry

Immunohistochemistry was performed using a combination of the microwave oven heating and the standard streptavidin–biotin–peroxidase complex (LSAB kit, Dako, Copenhagen, Denmark) methods. Briefly, slides were heated in 10 mM citrate buffer (pH 6.0) for six 5-min cycles for DCC and three 5-min cycles for $p21^{WAF1/Cip1}$, $p27^{Kip1}$ and Ki-67 antigen, using a microwave oven, and then incubated overnight at 4°C with optimum dilutions of primary antibodies. The antibodies employed were anti-human DCC monoclonal antibody (clone G97-449, × 100 dilution; Pharmingen, San Diego, CA, USA), anti-WAF1 monoclonal antibody for $p21^{WAF1/Cip1}$ (× 20 dilution, Novocastra Laboratories Ltd., Newcastle, UK), anti-Kip1 mouse monoclonal antibody for $p27^{Kip1}$ (× 1000 dilution; Transduction Laboratories, Lexington, KY, USA), and rabbit anti-human Ki-67 antigen (× 150 dilution; Dako). To confirm the specificity of binding, normal mouse or rabbit sera (× 500 dilution) were supplied instead of primary antibodies as negative controls.

### Scoring for immunoreactivity

Scoring of the immunohistochemistry results was made as previously reported (Saegusa et al, 1996). Briefly, based on the percentages of immunopositive epithelial cells in the totals of normal or neoplastic cells, subdivision for DCC and $p27^{Kip1}$ was into five categories as follows: 0, all negative; 1, < 10% positive cells; 2, 10–30%; 3, 30–50%; and 4, > 50%. Subclassification for $p21^{WAF1/Cip1}$ was as follows: 0, all negative; 1, < 5% positive cells; 2, 5–10%; 3, 10–30%; and 4, > 30%. The immunointensity was also subclassified into four groups: 0, negative; 1+, weak; 2+, moderate; and 3+, strong. Immunoreactivity scores were generated by multiplication of the values for the two parameters. Normal squamous or endocervical glandular epithelia adjacent to carcinomas in each case were used as positive controls for the three antibodies.

### Apoptotic and mitotic indices (AI and MI), and Ki-67 labelling index (LI)

Detection of apoptotic cells in haematoxylin and eosin stained sections was performed, using high-power (× 40 object and × 10 ocular) magnifications, in accordance with the criteria of Kerr et al (1994). AI values were calculated after examining at least 2000 nuclei in five randomly selected fields for each case. Areas of severe inflammatory cell infiltration and necrosis were excluded, since some doubtful cells were observed in such regions. MI and Ki-67 LI values were also calculated in a similar manner, counting either mitotic figures or Ki-67-positive nuclei.

### RT-PCR

Total cellular RNA was extracted from frozen tissues using Isogen (Nippon Gene Co., Tokyo, Japan) in accordance with the manufacturer's instructions. cDNA was synthesized from 5 μg of total RNA using RAV-2 reverse transcriptase (Takara, Shiga, Japan) in the presence of random primers (Takara) and a ribonuclease inhibitor (Takara) in a 20 μl reaction volume at 42°C for 60 min. One microlitre of cDNA solution was amplified by *Taq* polymerase (Takara) in a volume of 10 μl. For detection of DCC mRNA expression in exons 6–7, sense and antisense primers used were 5′-TTCCGCCATGGTTTTTAAATCA-3′, and 5′-AGCCTCATTTTCAGCCACACA-3′, corresponding to nucleotides 986–1007 and 1218–1198 respectively, in the cDNA sequences published by Fearon et al (1990). Other primer sets within exon 17 described by Reale et al (1994) were also applied: DCK2834S, 5′-CCCAGACTAACTGCATCATCATGAG-3′ (sense), and DCK3151A, 5′-CACCTACTGGTGGGAGCAT-3′ (anti-sense). For amplification of $p21^{WAF1/Cip1}$ mRNA, primers were 5′-GCGCCATGTCAGAACCGGCTG-3′ and 5′-GCAGGCTTCCTGTGGGCGGAT-3′, the products including the whole translated region of this gene (Nadal et al, 1997). The PCR procedure was performed with 35 cycles of denaturation at 94°C for 0.5 min. annealing at 55°C for 1 min and extension at 72°C for 1 min, with a final extension time of 7 min. As a negative control, water was supplied instead of template DNA for each examination. To examine the quality and quantity of the synthesized cDNA, amplification with β-actin specific primers (sense, 5′-TGCTATCCAGGCTGTGCTAT-3′ and antisense, 5′-GATGGAGTTGAAGGTAGTTT-3′) was also carried out. PCR assays were performed in duplicate or triplicate.

### SBH

A 10 μl aliquot of each PCR reaction mixture was electrophoresed in a 3.0% agarose gel and transferred to a Hybond N nylon membrane (Amersham, Tokyo Japan) with 10 × saline–sodium citrate (SSC) solution overnight. After prehybridization using DIG Easy Hyb (Boehringer Mannheim, Tokyo, Japan) solution, filters were hybridized overnight with each digoxigenin-labelled exon-specific probe, which corresponded to internal sites between the primer sets used. The sequences of oligonucleotide probes for DCC exon 6/7, DCC exon 17, $p21^{WAF1/Cip1}$ and β-actin were as follows: probe DCC exon 6/7 (5′-AATTGGATGAAGAATGGAGATGTGGTCATT-3′, encoding nucleotides 1087–1116 in the cDNA sequence), probe DCC exon 17 (5′-ATGAGTTGGACTCCTCCCTTGAAC-3′, nucleotides 2226–2250), probe $p21^{WAF1/Cip1}$ (5′-TGAGCGATGGAACTTCGACTTTGTCACCGA-3′, nucleotides 216–245), and probe β-actin (5′-ACTGACTACCTCATGAAGATCCTCACCGAG-3′, nucleotides 597–626). Hybridization signals were detected with a DIG Luminescent Detection Kit (Boehringer Mannheim). The conditions used for hybridization, washing and detection were in line with the manufacturer's recommendations. Between each hybridization the filter was stripped before being rehybridized with another probe.

Quantitation of hybridization signals for DCC exons 6/7 and 17, and β-actin, respectively, was performed by densitometric analysis using NIH Image software, version 1.58. The relative expression

© Cancer Research Campaign 1999

level of DCC mRNA was calculated by normalization to the hybridization signals for β-actin in each case.

### Western blot assay

Tissue samples were homogenized in 0.1 M phosphate-buffered saline (PBS) solution and clarified by centrifugation (12 000 rpm, 30 min). Fifty-microgram protein samples were separated by 12% sodium dodecyl sulphate (SDS)-polyacrylamide gel electrophoresis and then electroblotted (50 mA for 2 h) onto immobilon-P (Millipore, Tokyo, Japan). After blocking with Block Ace (Dainihonseiyaku Co., Osaka, Japan), the membranes were incubated overnight at 4°C with optimum dilutions of primary anti-WAF1 (× 20 dilution) and anti-Kip 1 (× 1000 dilution) mouse monoclonal antibodies. Reactivity was visualized using the Western Blot Chemiluminescence Reagent (NEN™ Life Science Products, Boston, MA, USA). As positive controls, normal squamous epithelium or squamous cell carcinomas (SCC) of uterine cervix were used since a high frequency of $p21^{WAF1/Cip1}$ expression has been demonstrated for the latter (Nadal et al, 1997) and $p27^{Kip1}$ immunopositivity is present in the former (Jordan et al, 1998).

### HPV detection

DNA samples were extracted from several serial 10 μm thick paraffin wax sections, through phenol–chloroform treatment. Aliquots of DNA (100 ng) were used as templates in a reaction volume of 10 μl containing 1 μM of each primer targeting the L1 open reading frame. The general primers used can detect eight types of HPV (6, 11, 16, 18, 30, 32 and 33) (Toh et al, 1992). The PCR assay entailed 40 cycles of 0.5 min at 94°C, 1 min at 40°C and 1 min at 72°C. The quality of the DNAs was confirmed with β-globin gene specific primers (Coates et al, 1991).

### Statistics

Data for AI, MI, Ki-67 LI and immunoreactivity scores of several markers were analysed using the Mann–Whitney *U*-test. Correlations were examined using the Pearson's correlation coefficient. The cut-off for statistical significance was defined as $P < 0.05$.

### RESULTS

### DCC expression

Moderate to strong cytoplasmic immunoreactivity for DCC was diffusely observed in endocervical glandular cells. The majority of other cervical components, including stromal cells, vessels and smooth muscular cells, lacked immunoreactivity (Figure 1A).

A variety of immunostaining patterns for DCC were found in AIS and AC lesions. Some tumours showed a diffuse strong cytoplasmic immunoreactivity, while others were negative or demonstrated sporadic positivity. Stromal tissues within tumours were consistently negative (Figure 1B,C).

The average values for DCC immunoreactivity scores were significantly lower in E-ACs than normal glandular cells, but similar values were found for normal, AIS and M-AC categories (Figure 2). No correlations with other clinical or pathological

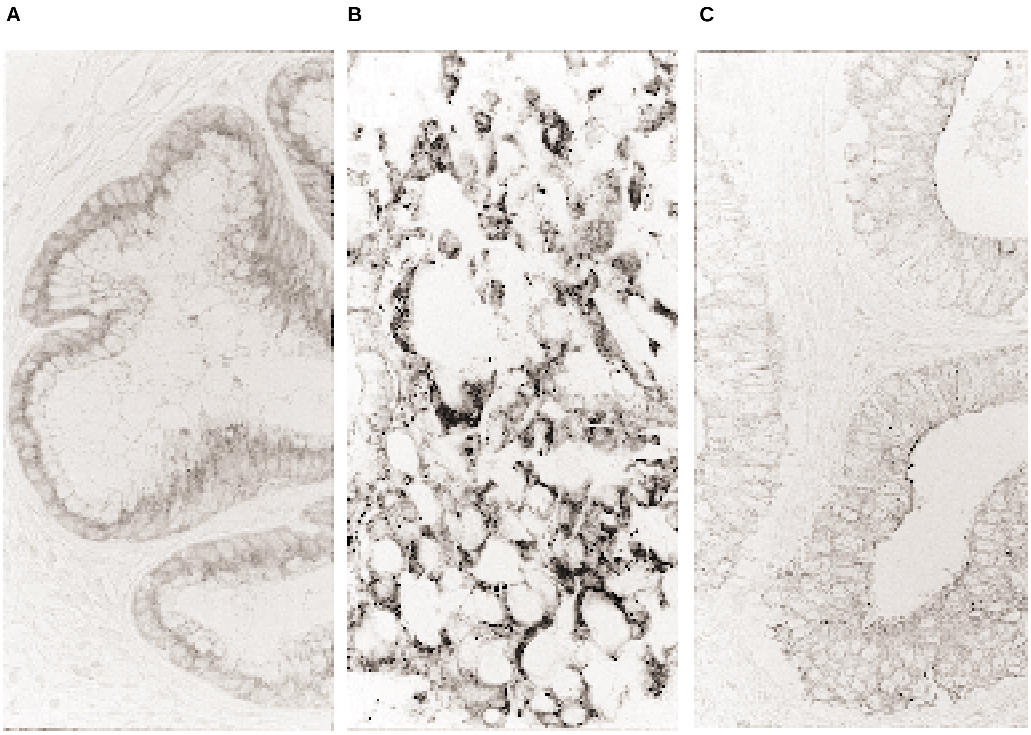

**Figure 1** DCC immunoreactivity in normal cervical glandular epithelium (**A**) and adenocarcinomas of mucinous (**B**) and endometrioid (**C**) types. Note the moderate (**A**), strong (**B**) and weak (**C**) immunointensities; × 400

© Cancer Research Campaign 1999

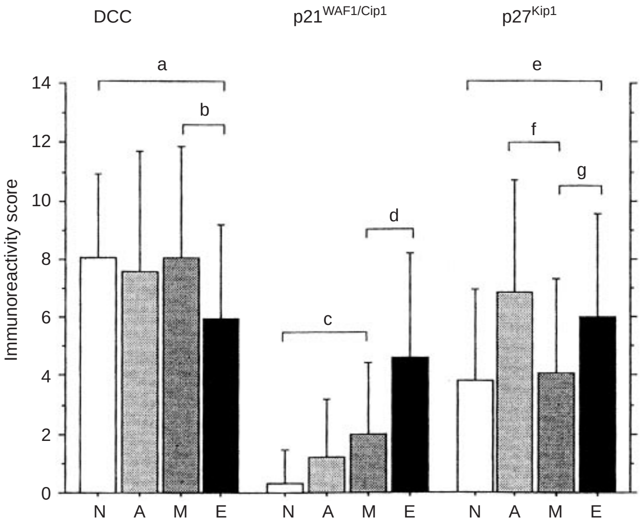

**Figure 2** Immunoreactivity scores for DCC, p21$^{WAF1/Cip1}$ and p27$^{Kip1}$. N, normal cervical glandular epithelium; A, adenocarcinoma in situ; M, mucinous type adenocarcinoma; E, endometrioid type adenocarcinoma. The data are means ± s.d. values. a, *P* = 0.012; b, *P* = 0.027; c, *P* = 0.0053; d, *P* = 0.0048; e, *P* = 0.012; f, *P* = 0.007; g, *P* = 0.0014

parameters, including histological malignancy, FIGO stage and lymph node status, were observed in ACs of either type (data not shown).

### p21$^{WAF1/Cip1}$ and p27$^{Kip1}$ expression

In normal cervical components, weak nuclear immunoreactivity for p21$^{WAF1/Cip1}$ was sporadically observed in a few glandular cells, while most stromal tissue components lacked staining. In contrast, moderate to strong immunopositivity for p27$^{Kip1}$ with distinct nuclear staining was occasionally found in epithelial and stromal elements, including infiltrating lymphocytes.

In AIS and ACs, clusters of p21$^{WAF1/Cip1}$-positive cells with weak to strong immunoreactivity were sporadically observed in tumour lesions, showing marked heterogeneity (Figure 3A,C). On the other hand, p27$^{Kip1}$-positive cells were diffusely distributed in most tumours (Figure 3B,D).

The average p21$^{WAF1/Cip1}$ scores increased in the sequence leading from normal cervical glandular cells to E-ACs, the difference being significant, while p27$^{Kip1}$ scores in AIS and E-ACs, respectively, were significantly higher than either normal or M-AC values (Figure 2). There was no association between either p21$^{WAF1/Cip1}$ or p27$^{Kip1}$ scores and any of the clinicopathological factors investigated (data not shown).

### AI, MI and Ki-67 LI

Detailed morphological features of apoptotic cells have been reported previously (Saegusa et al, 1996; Koshida et al, 1997). Cells undergoing apoptosis were more frequently identified in tumour lesions, in particular AIS and E-ACs, than in normal glandular epithelia. Similar findings for mitotic figures and nuclear Ki-67 immunopositivity were also observed (Figure 4). The average

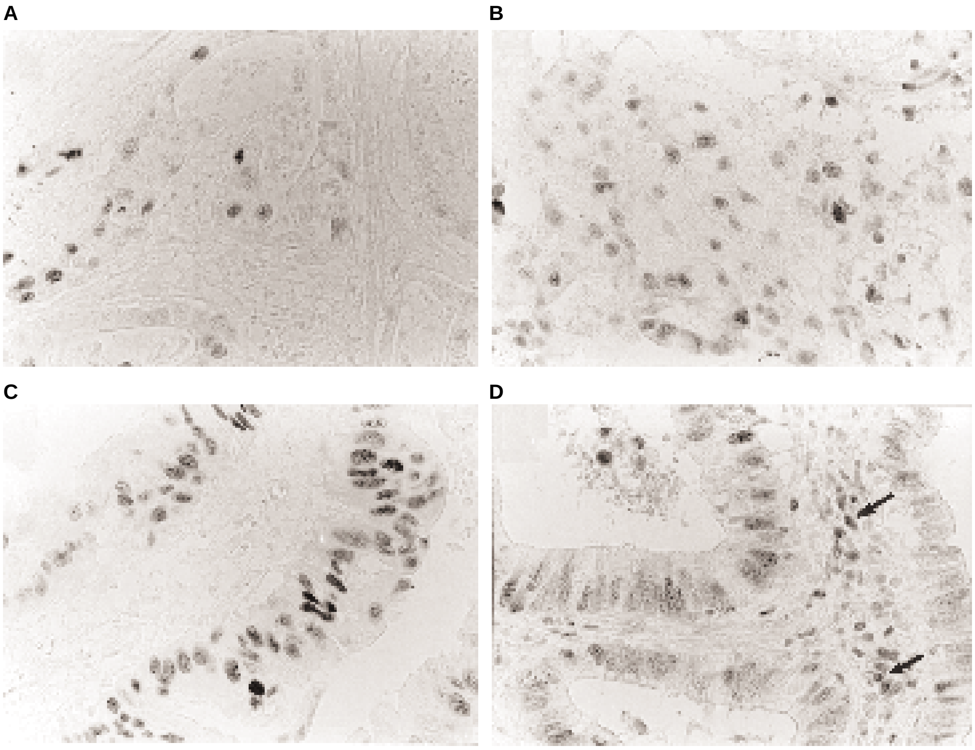

**Figure 3** Immunohistochemistry of p21$^{WAF1/Cip1}$ (**A**, **C**) and p27$^{Kip1}$ (**B**, **D**) in mucinous (**A**, **B**) and endometrioid (**C**, **D**) type cervical adenocarcinomas. Distinct nuclear staining of these proteins is evident. (**D**) Note the immunopositivity of infiltrating lymphocytes (indicated by arrows); × 400

 *© Cancer Research Campaign 1999*

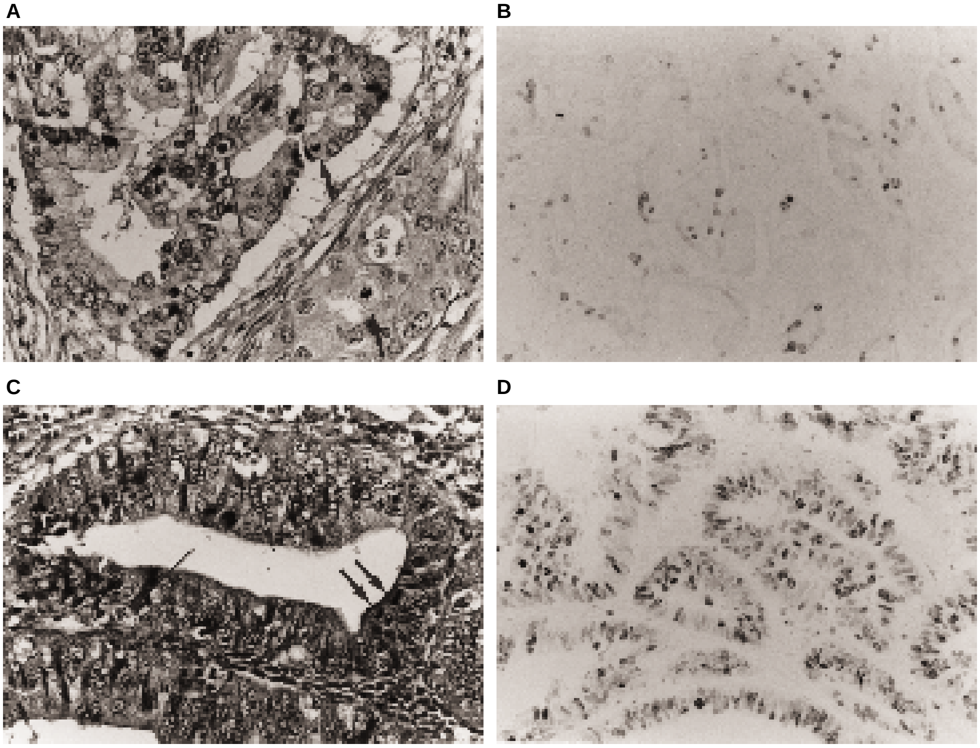

**Figure 4** (**A**, **C**) Apoptotic (indicated by long arrows) and mitotic cells (indicated by short arrows) in mucinous (**A**) and endometrioid (**C**) type adenocarcinomas. Apoptotic cells are characterized by overall shrinkage and homogeneously dark basophilic nuclei, presence of nuclear fragments (apoptotic bodies), sharply delineated cell borders surrounded by empty space, and homogeneous eosinophilic cytoplasm; haematoxylin and eosin × 400. (**B**, **D**) Ki-67 immunoreactivity in mucinous (**B**) and endometrioid (**D**) type adenocarcinomas; × 200

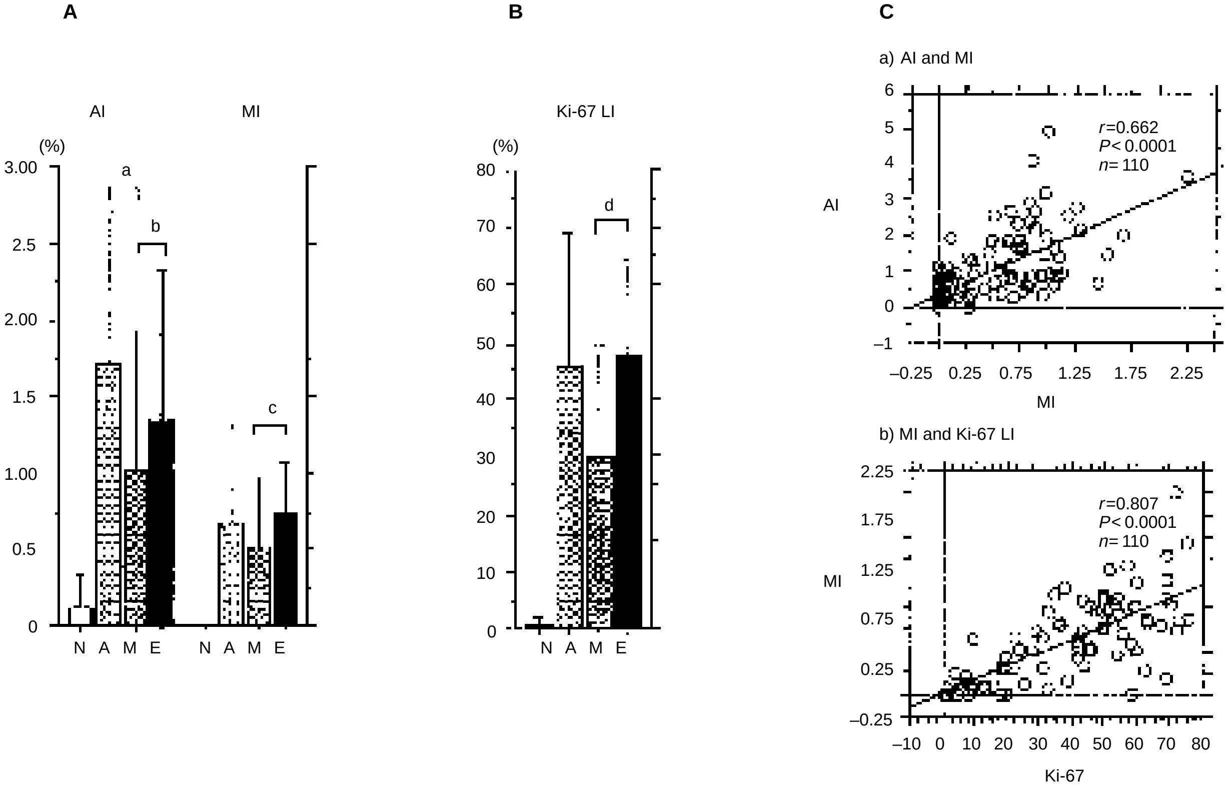

**Figure 5** (**A**) Apoptotic and mitotic indices (AI and MI); a, $P = 0.01$; b, $P = 0.04$; c, $P = 0.026$. (**B**) Ki-67 labelling index; d, $P = 0.02$. N, normal cervical glandular epithelium; A, adenocarcinoma in situ; M, mucinous type adenocarcinoma; E, endometrioid type adenocarcinoma. The data are means ± s.d. values. (**C**) Correlations among AI, MI and Ki-67 LI for all samples values of AI, as well as MI and Ki-67 LI values, were significantly lower in M-ACs than either AIS or E-ACs respectively (Figure 5A,B) Positive correlations among AI, MI and Ki-67 LI in normal and malignant lesions were noted (Figure 5C).

### Correlations among DCC, $p21^{WAF1/Cip1}$, $p27^{Kip1}$, apoptosis and cell proliferation

DCC scores was not correlated with values for $p21^{WAF1/Cip1}$ and $p27^{Kip1}$ in any category of cervical lesion. Furthermore, no association with AI, MI and Ki-67 LI was apparent (data not shown). By contrast, $p21^{WAF1/Cip1}$ and $p27^{Kip1}$, respectively, were positively correlated with Ki-67 LI (M-ACs vs $p21^{WAF1/Cip1}$, $r = 0.71$, $P < 0.0001$, and vs $p27^{Kip1}$, $r = 0.45$, $P = 0.01$; E-ACs vs $p21^{WAF1/Cip1}$, $r = 0.49$, $P = 0.005$, and vs $p27^{Kip1}$, $r = 0.41$, $P = 0.02$) but not AI and MI in both M- and E-AC lesions.

### RT-PCR/SBH assay

Amplicons with β-actin primers were detected with the expected molecular weight of 446 bp in all RNAs obtained from nine C-ACs samples, showing strong hybridization to the specific oligonucleotide probe.

RT-PCR products amplified by primer sets of either DCC exons 6–7 or 17 were observed with molecular weights of 233 bp and 341 bp respectively. A positive correlation ($r = 0.888$, $P = 0.0014$) between relative amounts of DCC exons 6–7 and 17 was noted. High DCC mRNA amounts (more than the average for ACs; exons 6–7, ≥ 0.7; exon 17, ≥ 0.6) were observed in three (33.3%) of nine ACs, in line with the high immunoreactivity scores (more than average values; M-ACs, ≥ 8; E-ACs, ≥ 6), with the exception of one case.

With the primer set for $p21^{WAF1/Cip1}$, the expected 571 bp fragment was amplified in five (55.6%) of nine ACs. Although unexpected fragments of approximately 350 bp were also observed, their significance was unclear. Of the positive cases, four were immunopositive. One case was negative for mRNA, but positive for immunoreactivity.

### Western blot assay

Western blot analysis of $p21^{WAF1/Cip1}$ revealed a molecular weight of 21 kDa in all samples investigated. An association between Western blot findings and either mRNA or immunohistochemical results was observed for four of five cases. For $p27^{Kip1}$, a 27 kDa band was detected in all five of the samples investigated, in line with the immunohistochemistry. Although several lower molecular weight bands were also observed, these may have been due to protein degradation.

### HPV detection

Specific amplicons using the primer set for the β-globin gene were observed in 64 (80%) of the 80 C-AC cases. HPV-DNAs were detected in 30 (46.9%) of these, including nine (39.1%) of 23 M-ACs, 12 (48%) of 25 E-ACs and nine (56.2%) of 16 AIS. There was no association between HPV infection and the histological phenotype. HPV-DNA positivity was significantly related to high values for $p27^{Kip1}$ scores ($P = 0.012$), AI ($P = 0.008$), MI ($P = 0.006$) and Ki-67 LI ($P < 0.0001$), but no association with DCC immunoreactivity was noted.

### DISCUSSION

The present immunohistochemical study demonstrated DCC expression in normal endocervical glandular cells with pronounced intracytoplasmic mucin accumulation, these being considered terminally differentiated and non-replicating cells. Previous studies revealed an association between DCC expression and cell differentiation in several tissues. For example, DCC is abundant in neurons of the central and peripheral nervous system, regarded as mature and non-dividing cells (Hedrick et al, 1994). In normal colonic epithelium, there are two major differentiated cell types, goblet cells and enterocytes, the expression being predominant in the former (Hedrick et al, 1994). Narayanan et al (1992) have also reported a functional role for DCC in the induction and maintenance of the differentiated phenotype of rat PC-12 cells derived from rat phaochromocytoma, in response to nerve growth factor. Our results, therefore, suggest that DCC expression may play an important role in controlling and maintaining the differentiation of cervical glandular cells.

One finding of interest in this context is that levels of DCC expression differed with the histological type of C-AC. With tumour progression from AIS lesions, DCC expression did not alter in M-ACs, while down-regulation was observed in E-ACs. Hedrick et al (1994) demonstrated earlier that high levels of DCC expression were observed in hyperplastic polyps and mucinous carcinomas, while the expression was decreased or absent in adenomatous polyps and adenocarcinomas of the colon, in line with less mucin production. This suggests that there are at least two pathways to colorectal tumorigenesis: DCC loss and failure to differentiate toward mucin-producing cells, and a mucinous type unassociated with alteration of DCC expression. We therefore conclude from the present data that in C-ACs, tumour histological phenotypes, in particular loss of the capacity to produce mucin, may be closely associated with change in DCC expression.

Up-regulation or de-regulation of $p21^{WAF1/Cip1}$ and $p27^{Kip1}$ may occur in a variety of human malignancies. For example, $p21^{WAF1/Cip1}$ expression is frequently observed in SCCs of the larynx, in association with tumour cell differentiation (Nadal et al, 1997). In gastric carcinomas, $p21^{WAF1/Cip1}$ expression is positively correlated with tumour stage, depth of invasion and lymph node metastasis, in line with a positive rather than an inverse association with Ki-67 positivity (Yasui et al, 1996). Recently, Sgambato et al (1997) demonstrated increased expression of $p27^{Kip1}$ in several cancer cell lines with rapid growth and high levels of cyclin E-associated kinase activity, suggesting the existence of a homeostatic feedback mechanism. Based on these reports, although it is widely accepted that $p21^{WAF1/Cip1}$ and $p27^{Kip1}$ act as negative regulators in normal cells (El-Deiry et al, 1993; Harper et al, 1993; Polyak et al, 1994; Toyoshima and Hunter, 1994), they may not be simply related to cell cycle progression in tumour cells.

In this study, significantly high values for AI, MI and Ki-67 LI were evident in E-ACs as compared with M-ACs, along with more pronounced expression of $p21^{WAF1/Cip1}$ and $p27^{Kip1}$. Considering the significant difference in DCC expression between the two histological phenotypes, it was expected that reduced DCC expression may contribute to rapid tumour tissue kinetics, since a close association between de-regulation of this gene and tumour progression or metastasis has been reported in several human malignancies (Kikuchi-Yanoshita et al, 1992; Enomoto et al, 1995; Kong et al, 1997; Goi et al, 1998). Our results, however, revealed that DCC expression was not related to any cell kinetic markers or

© Cancer Research Campaign 1999

clinicopathological factors. Further studies are necessary to clarify the association between DCC expression and tumour cell kinetics.

With regard to an association between mRNA- and protein-based assays, a positive correlation of DCC mRNA expression between exons 6–7 and 17 was evident in C-ACs, the amounts being positively associated with the immunoreactivity scores. Similar findings have also been reported for ovarian and endometrial carcinomas (Enomoto et al, 1995). With regard to p21$^{WAF1/Cip1}$ analysis, our results indicated similar positivity for this molecule among the three different methods in most cases. Nadal et al (1997) also demonstrated a significant correlation of p21$^{WAF1/Cip1}$ positivity between immunohistochemistry and mRNA assays in laryngeal SCCs. In contrast, although a concordance for Western blotting and immunohistochemistry of p27$^{Kip1}$ was observed in our series, the positivity in the former case may also include the expression of non-epithelial cell components, in particular infiltrating lymphocytes. Yasui et al (1997) have already reported that discrepancies in p27$^{Kip1}$ positivity between immunostaining and Western blotting findings for gastric carcinomas might be due to contamination of stromal cells expressing this protein.

It is widely accepted that E6 and E7 oncoproteins, produced by HPV, are able to bind to p53 or the retinoblastoma gene product (Rb), both well known as cell cycle-related proteins (Dyson et al, 1989; Werness et al, 1990), thereby blocking their negative effects on progression through the cell cycle. Our results also indicated that HPV infection is positively related to rapid tumour growth. Alteration of DCC expression, however, may be not affected.

In conclusion, the present study demonstrated that DCC expression in C-ACs is closely associated with mucinous differentiation but not changes in expression of p21$^{WAF1/Cip1}$ and p27$^{Kip1}$, apoptosis, cell proliferation and HPV infection.

### ACKNOWLEDGEMENT

We thank Ms M Hashimura for expert technical assistance.

### REFERENCES

Coates PJ, d'Ardenne AJ, Khan G, Kangro HO and Slavin G (1991) Simplified procedures for applying the polymerase chain reaction to routinely fixed paraffin wax sections. *J Clin Pathol* **44**: 115–118

Dyson N, Howley PM, Munger K and Harlow E (1989) The human papilloma virus-16 E7 oncoprotein is able to bind to the retinoblastoma gene product. *Science* **243**: 3812–3815

El-Deiry WS, Tokino T, Velculescu VE, Levy DB, Parsons R, Trent JM, Lin D, Mercer WE, Kinzier KW and Vogelstein B (1993) WAF1, a potential mediator of p53 tumor suppression. *Cell* **75**: 817–825

Enomoto T, Fujita M, Cheng C, Nakashima R, Ozaki M, Inoue M and Nomura T (1995) Loss of expression and loss of heterozygosity in the DCC gene in neoplasms of the human female reproductive tract. *Br J Cancer* **71**: 462–467

Fearon ER, Cho KR, Nigro JM, Kern SE, Simons JW, Ruppert JM, Hamilton SR, Preisinger AC, Thomas G, Kinzler KW and Vogelstein B (1990) Identification of a chromosome 18q gene that is altered in colorectal cancers. *Science* **247**: 49–56

Gao X, Honn KV, Grignon D, Sakr W and Chen YQ (1993) Frequent loss of expression and loss of heterozygosity of the putative tumor suppressor gene DCC in prostatic carcinomas. *Cancer Res* **53**: 2723–2727

Gima T, Kato H, Honda T, Imamura T, Sasazuki T and Wake N (1994) DCC gene alteration in human endometrial carcinomas. *Int J Cancer* **57**: 480–485

Ginsberg D, Mechta F, Yaniv M and Oren M (1991) Wild-type p53 can down-modulate the activity of various promoters. *Proc Natl Acad Sci USA* **88**: 9979–9983

Goi T, Yamaguchi A, Nakagawara G, Urano T, Shiku H and Furukawa K (1998) Reduced expression of deleted colorectal carcinoma (DCC) protein in established colon cancers. *Br J Cancer* **77**: 466–471

Hannon GJ and Beach D (1994) p15$^{INK4B}$ is a potent effector of TGF-β-induced cell cycle arrest. *Nature* **371**: 257–261

Harper JW, Adami GR, Wei N, Keyomarski K and Elledge SJ (1993) The p21 Cdk-interacting protein Cip1 is a potent inhibitor of G1 cyclin-dependent kinases. *Cell* **75**: 805–816

Hedrick L, Cho KR, Fearon ER, Wu T-C, Kinzler KW and Vogelstein B (1994) The DCC gene product in cellular differentiation and colorectal tumorigenesis. *Genes Dev* **8**: 1174–1183

Huang Y, Boynton RF, Blount PL, Silverstein RJ, Yin J, Tong Y, McDaniel TK, Newkirk C, Resau JH, Sridhara R, Reid BJ and Meltzer SJ (1992) Loss of heterozygosity involves multiple tumour suppressor genes in human esophageal cancers. *Cancer Res* **52**: 6525–6530

Jordan RCK, Bradley G and Slingerland J (1998) Reduced levels of the cell-cycle inhibitor p27$^{Kip1}$ in epithelial dysplasia and carcinoma of the oral cavity. *Am J Pathol* **152**: 585–590

Kamb A, Gruis NA, Weaver-Feldhaus J, Liu Q, Harsham K, Tavtigian SV, Stockert E, Day RS, III, Johnson BE and Scholinck MHA (1994) A cell cycle regulator potentially involved in genesis of many tumor types. *Science* **264**: 436–440

Kerr JFR, Winterford CM and Harmon BV (1994) Apoptosis: its significance in cancer and cancer therapy. *Cancer* **73**: 2013–2026

Kikuchi-Yanoshita R, Konishi M, Fukunari H, Tanaka K and Miyaki M (1992) Loss of expression of the DCC gene during progression of colorectal carcinomas in familial adenomatous polyposis and non-familial adenomatous polyposis patients. *Cancer Res* **52**: 3801–3803

Klingelhutz AJ, Hedrick L, Cho KR and McDougall JK (1995) The DCC gene suppresses the malignant phenotype of transformed human epithelial cells. *Oncogene* **10**: 1581–1586

Kong X-T, Choi SH, Inoue A, Xu F, Chen T, Takita J, Yolota J, Bessho F, Yanagisawa M, Hanada R, Yamamoto K and Hayashi Y (1997) Expression and mutational analysis of the DCC, DPC4, and MADR2/JV18-1 genes in neuroblastoma. *Cancer Res* **57**: 3772–3778

Koshida Y, Saegusa M and Okayasu I (1997) Apoptosis, cell proliferation and expression of Bcl-2 and Bax in gastric carcinomas: immunohistochemical and clinicopathological study. *Br J Cancer* **75**: 367–373

Mercer WE, Shields MT, Lin D, Appella E and Ullrich SJ (1991) Growth suppression induced by wild-type p53 protein is accompanied by selective down-regulation of proliferating-cell nuclear antigen expression. *Proc Natl Acad Sci USA* **88**: 1958–1962

Nadal A, Jares P, Cazorla M, Fernandez PL, Sar Juan X, Hernandez L, Pinyol M, Aldea M, Mallofre C, Muntane J, Traserra J, Campo E and Cardesa A (1997) p21$^{WAF1/Cip1}$ expression is associated with cell differentiation but not with p53 mutations in squamous cell carcinomas of the larynx. *J Pathol* **183**: 156–163

Narayanan R, Lawlor RQJ, Schaapveld KR, Cho KR, Vogelstein B, Tran PBV, Osborne MP and Telang NT (1992) Antisense RNA to the putative tumor suppressor gene DCC transforms Rat-1 fibroblasts. *Oncogene* **7**: 553–561

Nobori T, Miura K, Wu DJ, Lois A, Takabayashi K and Carson DA (1994) Deletions of the cyclin-dependent kinase-4 inhibitor gene in multiple human cancers. *Nature* **368**: 753–756

Park DJ, Wilczynski SP, Paquette RL, Miller CW and Koeffler HP (1994) p53 mutations in HPV-negative cervical carcinoma. *Oncogene* **9**: 205–210

Polyak K, Kato JY, Solomon MJ, Sherr CJ, Massague J, Roberts JM and Koff A (1994) p21KIP1, a cyclin-Cdk inhibitor, links transforming growth factor-β and contact inhibition to cell cycle arrest. *Genes Dev* **8**: 9–22

Reale MA, Hu G, Zafar AI, Getzenberg RH, Levine SM and Fearon ER (1994) Expression and alternative splicing of the deleted in colorectal cancer (DCC) gene in normal and malignant tissues. *Cancer Res* **54**: 4493–4501

Saegusa M, Takano Y, Hashimura M, Shoji Y and Okayasu I (1995) The possible role of bcl-2 expression in the progression of tumors of the ureine cervix. *Cancer* **76**: 2297–2303

Saegusa M, Kamata Y, Isono M and Okayasu I (1996) Bcl-2 expression is correlated with a low apoptotic index and associated with progesterone receptor immunoreactivity in endometrial carcinomas. *J Pathol* **180**: 275–282

Serrano M, Hannon GJ and Beach D (1993) A new regulatory motif in cell-cycle control causing specific inhibition of cyclin D/CDK4. *Nature* **366**: 704–707

Sgambato A, Zhang Y-J, Arber N, Hibshoosh H, Doki Y, Ciaparrone M, Santella RM, Cittadini A and Weinstein IB (1997) Deregulated expression of p27$^{Kip1}$ in human breast cancers. *Clin Cancer Res* **3**: 1879–1887

Toh Y, Kuwano H, Tanaka S, Baba K, Matsuda H, Sugimachi K and Mori R (1992) Detection of human papillomavirus DNA in esophageal carcinoma in Japan by polymerase chain reaction. *Cancer* **70**: 2234–2238

Toyoshima H and Hunter T (1994) p27, a novel inhibitor of G1 cyclin-Cdk protein kinase activity, is related to p21. *Cell* **78**: 67–74

© Cancer Research Campaign 1999

Uchino S, Tsuda H, Noguchi M, Yokota J, Terada M, Saito T, Kobayashi M, Sugimura T and Hirohashi S (1992) Frequent loss of heterozygosity at the DCC locus in gastric cancer. *Cancer Res* **52**: 3099–3102
Werness BA, Levine AJ and Howley PM (1990) Association of human papillomavirus types 16 and 18 Eˆ proteins with p53. *Science* **248**: 76–79
Yasui W, Akama Y, Kuniyasu H, Yokozaki H, Semba S, Shimamoto F and Tahara E (1996) Expression of cyclin-dependent kinase inhibitor $p21^{WAF1/CIP1}$ in non-neoplastic mucosa and neoplasia of the stomach: relationship with p53 status and proliferative activity. *J Pathol* **180**: 122–128
Yasui W, Kudo Y, Semba S, Yokozaki H and Tahara E (1997) Reduced expression of cyclin-dependent kinase inhibitor $p27^{Kip1}$ is associated with advanced stage and invasiveness of gastric carcinomas. *Jpn J Cancer Res* **88**: 625–629

 *© Cancer Research Campaign 1999*
